# The cost of adaptability: resource availability constrains functional stability under pulsed disturbances

**DOI:** 10.1128/msphere.00727-23

**Published:** 2024-01-11

**Authors:** Angel Rain-Franco, Hannes Peter, Guilherme Pavan de Moraes, Sara Beier

**Affiliations:** 1 UMR 7621 Laboratoire d’Océanographie Microbienne, Observatoire Océanologique de Banyuls-sur-Mer, Sorbonne Université, Banyuls-sur-Mer, France; 2 Department of Biological Oceanography, Leibniz Institute for Baltic Sea Research Warnemünde, Rostock, Germany; 3 River Ecosystems Laboratory, Ecole Polytechnique Federale de Lausanne, Lausanne, Switzerland; 4 Department of Botany, Graduate Program in Ecology and Natural Resources (PPGERN), Laboratory of Phycology, Universidade Federal de São Carlos, São Carlos, Brazil; University of Wisconsin-Madison, Madison, Wisconsin, USA

**Keywords:** resistance, resilience, community traits, continuous culture, life history

## Abstract

Global change exposes ecosystems to changes in the frequency, magnitude, and concomitancy of disturbances, which impact the composition and functioning of these systems. Here, we experimentally evaluate the effects of salinity disturbances and eutrophication on bacterial communities from coastal ecosystems. The functional stability of these communities is critically important for maintaining water quality, productivity, and ecosystem services, such as fishery yields. Microbial functional stability can be maintained via resistance and resilience, which are reflected in genomic traits such as genome size and codon usage bias and may be linked to metabolic costs. However, little is known about the mechanisms that select these traits under varying nutrient regimes. To study the impact of pulsed disturbances on community assembly and functioning depending on metabolic costs, we performed a 41-day pulse disturbance experiment across two levels of resource availability. Our setup triggered stochastic community re-assembly processes in all treatments. In contrast, we observed consistent and resource availability-dependent patterns of superordinate community functioning and structural patterns, such as functional resistance in response to disturbances, genomic trait distributions, and species diversity. Predicted genomic traits reflected the selection for taxa possessing resistant- and resilience-related traits, particularly under high nutrient availability. Our findings are a step toward unraveling the compositional and genomic underpinnings of functional resistance in microbial communities after exposure to consecutive pulse disturbances. Our work demonstrates how resource availability alleviates metabolic constraints on resistance and resilience, and this has important consequences for predicting water quality and ecosystem productivity of environments exposed to global change.

**IMPORTANCE:**

Understanding the communities’ responses to disturbances is a prerequisite to predicting ecosystem dynamics and, thus, highly relevant considering global change. Microbial communities play key roles in numerous ecosystem functions and services, and the large diversity, rapid growth, and phenotypic plasticity of microorganisms are thought to allow high resistance and resilience. While potential metabolic costs associated with adaptations to fluctuating environments have been debated, little evidence supports trade-offs between resource availability, resistance, and resilience. Here, we experimentally assessed the compositional and functional responses of an aquatic microbial model community to disturbances and systematically manipulated resource availability. Our results demonstrate that the capacity to tolerate environmental fluctuations is constrained by resource availability and reflected in the selection of genomic traits.

## INTRODUCTION

There is mounting evidence that anthropogenic global change rapidly alters natural disturbance regimes, with important consequences for ecosystems and the services they provide (1–3). Particularly, climate warming-induced droughts and heavy precipitation events affect both terrestrial and aquatic ecosystems (4–6). Concomitantly, land-use changes, such as intensified freshwater use for irrigation or eutrophication linked to fertilizer application, deteriorate water quality and raise concerns for critical ecosystem services (7–9). Coastal areas are particularly vulnerable to such combined disturbances (7). While interactions between isolated disturbance events, biodiversity, and ecosystem consequences have been elucidated (e.g., 10, 11), the underlying traits that allow populations to resist or recover from multiple and consecutive disturbances remain less studied. Although ecological theory exists on the effects of consecutive disturbances (1), this has been developed mainly for larger organisms with long generation times and complex life-histories, while we currently lack an understanding of the effects of frequent disturbances on microbial communities. This is in stark contrast with the important role played by these microbial communities in water quality and ecosystem productivity, notably in coastal ecosystems (12) .

Disturbance ecology and biodiversity–ecosystem functioning research have highlighted the importance of species diversity for ensuring functional resistance and resilience (10, 11). The response of microbial communities to disturbances is thought to be determined by the degree to which a community is insensitive to a disturbance, its resistance, and the rate at which a community returns to the pre-disturbance state, its resilience (13). While functionally redundant taxa can contribute to the resistance and resilience of microbial communities (i.e., insurance effect of diversity), response traits allow individual populations to adapt to and recover from disturbances (14, 15). Of particular interest are life-history traits associated with stress tolerance and adaptation (i.e., resistance traits) as well as traits related to growth and reproduction (i.e., resilience traits) (16). In line with these expectations, previous experimental work has linked the functional stability of microbial communities to the presence of generalist taxa, which exhibit increased resistance against disturbances due to their broad niche (17).

It has been suggested that traits inferred from the genomic information of microbial communities approximate life history relevant for resistance and resilience (18). Genomic traits such as for instance genome size and the fraction of transcription factors (%TF) were linked to classifications along the generalist–specialist continuum because additional auxiliary genes and regulatory capacity enhance aptness to environmental change (19, 20). For example, the expression of genes encoding the transport of osmolytes has been demonstrated to increase species fitness under changing salinity conditions (15), and larger genomes are more likely to possess such genes encoding adaptive responses. In contrast, codon usage bias and the number of 16S rRNA gene copies (RRN) are related to maximal growth rate and lag-phase duration (21, 22). Both fast growth rates and short lag phases are traits associated with resilience after disturbances, but these traits usually come at the cost of reduced resource use efficiency (23, 24). Similarly, resistance traits can be associated with metabolic costs. Such costs may be related to the strategy of generalists to process more environmental information than specialists and regulate their responses accordingly (25).

Microbial communities have been shown to allocate available resources either toward resistance or resilience, with oligotrophic environments favoring resistant but slowly recovering communities, while the opposite is true for nutrient-rich environments (e.g., see reference 26). However, in contrast to such resistance–resilience trade-off, oligotroph aquatic bacterial taxa like SAR11 have been described as slow-growing organisms with limited physiological and metabolic flexibility that are consequently sensitive to environmental change (27). While literature consistently reports that oligotroph environments select for slow growing and consequently less resilient prokaryotes, these conditions may accordingly favor prokaryotes with either high or low resistance.

To address these conflicting observations on the trade-off between resistance and resilience, a recent study investigated trait–trait variations including the above-highlighted genomic traits as markers for resilience and resistance from ~18,000 bacterial genomes (18). The observed trait–trait variations confirmed the hypothesized negative correlation (i.e., trade-off) between resistance- and resilience-related genomic traits only in prokaryotes with genomes > 5 Mbp, which are typically found in soil environments. In contrast, for taxa with genome sizes that are typical for aquatic prokaryotes (<4 Mbp), resistance- and resilience-related genomic traits correlated positively. This *in silico* analysis suggests that the addition of nutrients to aquatic environments would lead to a simultaneous increase of both resistance and resilience.

Here, by leveraging continuous cultivation experiments with complex aquatic bacterial communities, we address the hypothesis that exposure to multiple consecutive salinity pulse disturbances simultaneously selects for resistant and resilient community members if the community has sufficient nutrient supply. We posit that this selection imposed by consecutive disturbances manifests in non-stochastic community re-assembly and is reflected in genomic traits associated with resistance and resilience. To unravel trade-offs between resistance, resilience, and resource availability, we contrasted two levels of resource availability. Furthermore, we monitored bacteria production (BP) and respiration to derive bacterial growth efficiency (BGE). Our findings highlight the importance of genomic traits in explaining community responses to consecutive disturbances. We provide experimental evidence for constraints of resource availability on resistance and resilience, which is critically important considering multiple and simultaneous global changes.

## RESULTS AND DISCUSSION

**Fig 1:**
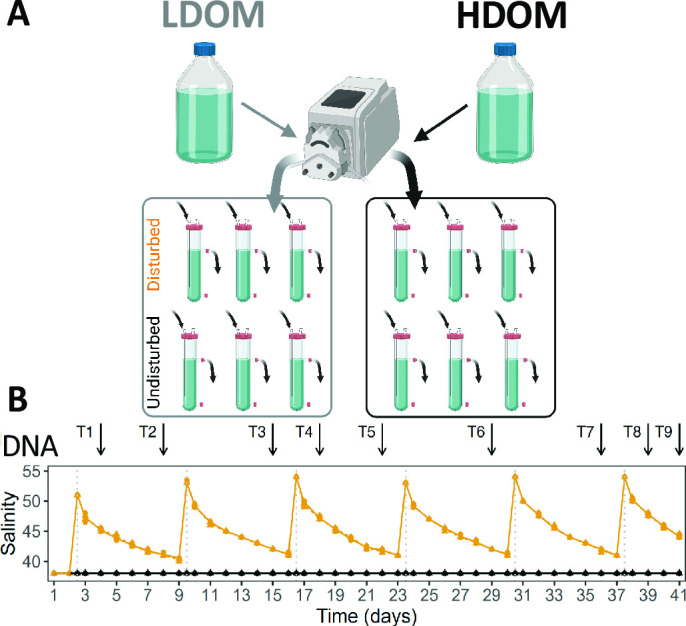
Experimental setup. (**A**) Graphical schema summarizing the main experimental conditions and (B) salinity induced-pulses and the DNA sampling frequency.

**Fig 2:**
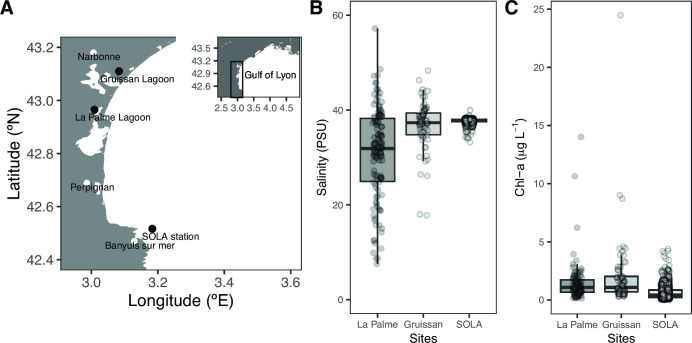
Sampling locations and environmental characteristics of the sample sites, from which the starting inocula were obtained. (**A)** Geographic locations of the La Palme and Gruissan lagoons as well as the Mediterranean coastal SOLA field station. (**B**) Temporal salinity (PSU) and (**C**) chlorophyll-a (μg L^–1^) variability. Time-series environmental data for La Palme (23 September 1989–11 September 2020, *n* = 206) and Gruissan (23 September 1989–08 August 2019, *n* = 128) were obtained from https://wwz.ifremer.fr/surval. Environmental data for the SOLA station were provided by the SOMLIT program (https://www.somlit.fr/; 04 January 2005–24 November 2020, *n* = 1443).

We performed a 41-day continuous culture experiment in a chemostat with repeated pulse disturbances and undisturbed control treatments across two resource availability levels (Fig. 1A). The baseline salinity level of all incubations was set to 38 psu and approximately reflected the salinity in our reference systems during sampling (Table S1). Weekly pulse disturbances were induced by adding a saturated NaCl solution, which resulted in a salinity increase of ~13 psu in the disturbed treatments. This reflects the amplitude of salinity disturbance that occurs in some of our reference systems (Fig. 2B; Fig. S1). The constant inflow of fresh medium washed out the added salt, thereby leading to salinity pulses. In total, the communities were exposed to six pulses. Resource availability was manipulated by two different sources of dissolved organic matter (DOM) representing low and high nutrient levels (oDOM and eDOM, respectively). As expected, differences in nutrient availability were reflected in different cell abundances with 0.8 ± 0.4 × 10^6^ cells mL^−1^ (oDOM) and 2.1 ± 0.6 × 10^6^ cells mL^−1^ (eDOM), respectively. These values represent typical cell densities for oligotrophic (oDOM) or mesotrophic to eutrophic (eDOM) aquatic environments.

We opted for a “super-diverse” metacommunity as the inoculum such that selection during experimental disturbances could act on a rich assemblage with complementary traits. The cultures were therefore inoculated with a metacommunity composed of microbial communities from several coastal Mediterranean marine and lagoon sites that differed in their disturbance history and eutrophication level (Fig. 2).

### Community composition and assembly

16S rRNA gene amplicon sequencing showed similar trends across treatments. Early experimental stages were dominated by members of Flavobacteriales and Enterobacterales (Fig. S2). At intermediate stages, Rhodobacterales and Caulobacterales increased in abundance. At the end of the experiment, diverse taxonomies were observed even among replicates, including, for instance, members of the Caulobacterales, Sphingomonadales, Rhodospirillales, and Enterobacterales.

**Fig 3:**
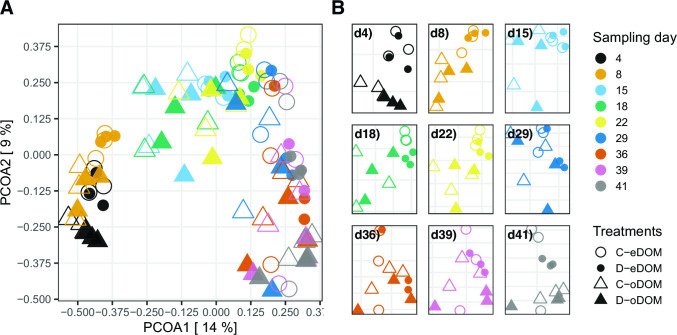
Community structures. (**A)** Overview of principal coordinate analysis (PCoA) biplot including all data points (Bray–Curtis distances). (**B)** PCoAs (Bray–Curtis distances) for individual sampling days; axes of the individual plots are differently scaled as indicated by the grid lines.

Permutational multivariate analysis of variance (PERMANOVA), based on amplicon sequence variants (ASVs), suggested that resource availability was the strongest structuring factor of community composition (F = 30.09, *P* ≤ 0.001), followed by the incubation time (F = 14.2, *P* ≤ 0.001) and then the disturbance regime (F = 3.3, *P* = 0.008; Fig. 3; Table S2). These observations point to a pronounced and treatment-influenced succession during the course of the experiment, and high taxon turnover was supported by a Bray–Curtis distance of ≥0.72 between the first and the last sample (T1 vs T9, Fig. 3B). Considerable divergence in community compositional patterns among replicated treatments, both at the ASV and order level (Fig. S2), indicated a pronounced stochastic compositional re-assembly component. Analyses of beta nearest taxon indices pointed to a treatment-independent prevalence of stochastic rather than deterministic events during community re-assembly (Fig. S3). Our findings thereby contrast with results from a continuous culture study in which periodical disturbance induced a shift toward a more deterministic assembly of microbial communities (28). However, another continuous culture experiment suggested that high species diversities in the starting communities increase the contribution of stochastic events during community assembly (29). The highly diverse initial metacommunity inoculum possibly in combination with missing dispersal among treatments (30) may have contributed to stochastic re-assembly processes in our cultures.

Despite the stochasticity in community re-assembly, more deterministic selection may have shaped trait distributions. Both bacterial life-history traits and the above-highlighted resistance- and resilience-related genomic features are phylogenetically conserved (18, 31). Phylogenetic distance-based community composition should accordingly reflect a greater impact of the disturbance regime compared to the purely compositional metric applied above. Indeed, PERMANOVA based on a phylogenetic distance metric showed that the disturbance regime had a greater impact than in the analysis based solely on the community composition (F = 5.9, *P* = 0.009; Fig. S4; Table S2). This indicates that repeated pulse disturbances lead to more trait-based and consequently functional similarities between communities, with only moderate overlap in taxonomic composition.

### Community functional resistance

In order to assess community functional responses to disturbances, a resistance index was computed by quantifying the relative change of BP, respiration, and BGE before and after each disturbance compared to the corresponding activity change in the respective control treatments.

**Fig 4:**
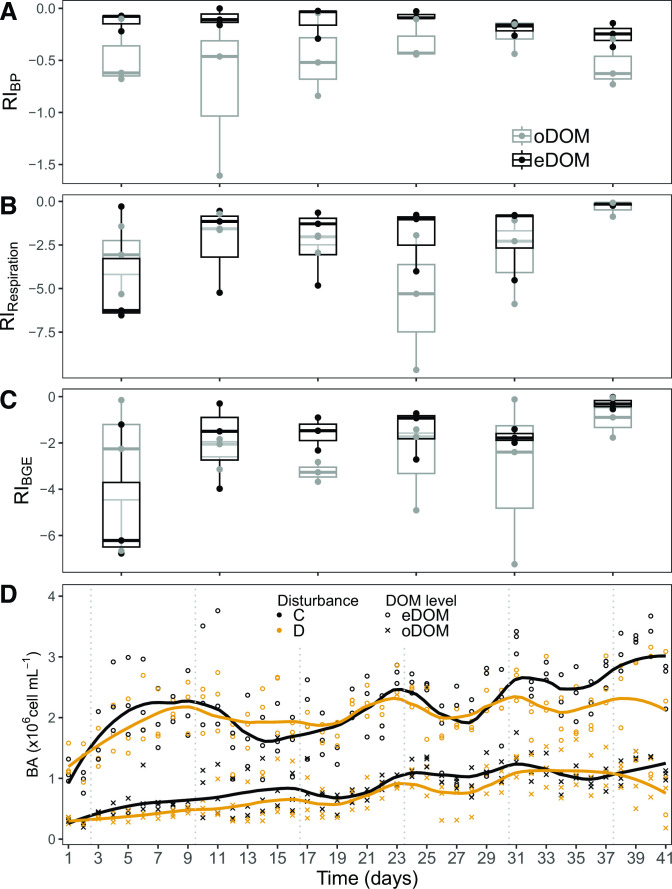
Community functional parameters. Resistance indices for (**A**) bacterial production (BP), (**B**) respiration, and (**C**) BGE. (**D**) Bacterial abundance (BA), lines indicate locally fitted values (loess smoothing) under each disturbance and DOM regime.

We found that bacterial communities without resource limitation (eDOM) exhibited significantly higher BP resistance than communities exposed to resource limitation (oDOM) (Fig. 4A; Table 1). More noticeably, patterns of BP were manifested in cell abundance throughout the experiment. Specifically, salinity disturbances led to low resistance of BP in communities grown under resource limitations (oDOM), causing a significant decrease in cell abundance. In contrast, BP was highly resistant and cell numbers remained unaffected by disturbances under alleviated resource conditions (eDOM) (Table 1; Fig. 4D). These findings highlight the role of resource availability in the resistance of aquatic microbial communities: we posit that resource abundance can alleviate metabolic costs associated with resistance (adaptability) and fundamentally alter the functional consequences of disturbances. This has important consequences considering multiple and simultaneous perturbations associated with global change, including widespread eutrophication.

**TABLE 1:** Results of statistical tests to evaluate the effect of nutrient availability on functional resistance^a^

	oDOM	eDOM	rmANOVA				LMM_oDOM_	LMM_eDOM_
RIs bulk functioning	Mean	Mean	F_Time_	P_Time_	F_DOM_	P_DOM_	Slope	Slope
Bacterial production	−0.4	−0.2	0.56	0.726	10.83	0.003**	0.04	−0.03
Respiration	−1.7	−1.4	2.13	0.098•	0.41	0.527	0.17	0.63*
Bacterial growth efficiency	−0.9	−0.4	1.72	0.170	0.92	0.347	0.24	0.66*

^a^ *•P* < 0.1; *P < 0.05; **P < 0.01.

Interestingly, we could not detect significant temporal trends in BP resistance (Fig. 4A; Table 1). Consecutive disturbances did not cause a steady increase of functionally resistant community members over time. Instead, the observed differences between the eDOM and oDOM treatments seemed inherent to the respective communities grown under different nutrient regimes, with differences in cell densities between disturbed and control oDOM regimes already observed after the first disturbance (Fig. 4D).

Temporal patterns of resistance for respiration and BGE differed from those observed for BP. The results suggest, at least in the eDOM incubations, an increase in resistance over time in response to consecutive disturbances. Conversely, neither respiration nor growth efficiency differed significantly between treatments with (oDOM) or without (eDOM) resource limitation (Table 1). We attribute this to the effect of the maintenance metabolism on bacterial respiration, which is independent of resource availability. Taken together, the results point to different mechanisms that determine the resistance of BP and respiration.

### Genomic trait distributions and consequences on diversity patterns

**Fig 5:**
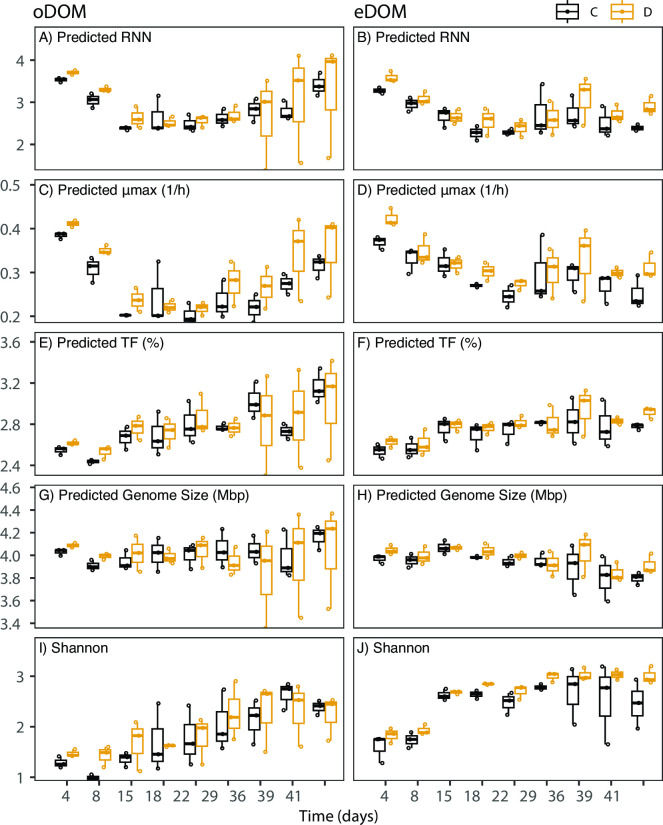
Boxplots displaying community-weighted means (CWMs) of predicted genomic traits. oDOM and eDOM are displayed in the left and right panels, respectively. (**A, B**) Number of 16S rRNA gene copies (RRN), (**C, D**) maximal growth rates (μmax), (**E, F**), fraction of transcription factors (%TF), (**G**, **H**) genome size, and (**I**, **J**) Shannon diversity index.

To explore genomic traits underlying the observed functional resistance, response trait values for genome size and %TF were extrapolated for individual ASVs from values for close relatives present in databases. Likewise, we also extrapolated trait values for maximal growth rate estimates delineated from codon usage biases and RRN as proxies for the resilience of individual community members represented by ASVs. This allowed us to compute community-weighted means (CWMs, mean trait values weighted for ASV relative abundances) of the predicted genomic traits in individual communities. CWMs of all predicted genomic traits with exception of genome size changed significantly over time and independently of the disturbance regime or resource availability (Fig. 5; Table 2). Roughly constant CWM of genome sizes (Fig. 5G and H), reflect BP resistances that differed in response to nutrient availability but remained stable over time (Fig. 4A). This is particularly noteworthy when considering the pronounced successional turnover, indicating that resource limitation may exert stronger controls on the functional consequences of disturbances than community composition or diversity. This is key when devising potential management strategies.

In contrast, temporal dynamics of genomic trait distributions were evident for resilience-related traits, with a pronounced decrease in the predicted growth rates, as well as a decrease in the predicted RRN after the second experimental week. In line with these observations, high RRNs have been described during the early successional stages of microbial communities from different environments (32, 33).

**TABLE 2:** Results of the statistical test to evaluate the effect of disturbance on predicted genomic trait distributions^*a*^

					rmANOVA				One-tailed test
		Trait^*c*^	Mean_C_	Mean_D_	F_Time_	P_Time_	^*b*^F_C/D_	*P*_C/D_	*P*_C/D_
oDOM	Resilience	RRN	2.86	2.93	3.57	0.004***	0.27	0.608	0.138
		µmax^†^	0.27	0.30	14.54	0.000***	8.43	0.006*	0.002*
	Resistance	%TF	2.76	2.78	5.25	0.000***	0.12	0.737	0.334
		Genome size	4.02	3.99	0.34	0.943	0.17	0.685	0.753
	Diversity	Shannon	1.81	1.90	8.45	0.000***	0.70	0.409	NA
eDOM	Resilience	RRN	2.64	2.84	7.26	0.000***	5.98	0.020*	0.007*
		µmax^†^	0.29	0.32	6.79	0.000***	8.13	0.007*	0.001*
	Resistance	%TF	2.72	2.79	4.73	0.001*	4.02	0.053•	0.001*
		Genome size	3.93	3.98	3.37	0.006*	3.80	0.060•	0.008*
	Diversity	Shannon	2.39	2.67	16.10	0.000***	14.60	0.001*	NA

^*a*^ •*P* < 0.1;*P < 0.05; ***P < 0.001.

^*b*^ C: control; D: disturbed treatment.

^*c*^ †: maximal growth rates

Besides the predominance of stochastic species-level assembly processes that occurred independent of the disturbance regime, consistent disturbance-driven effects were apparent for trait distributions. Disturbances impacted the distribution of predicted genomic traits according to resource availability, generally following our predictions. In the absence of resource limitation (eDOM), all evaluated genomic traits (genome size, %TF; RRN, maximal growth rates) differed significantly between the disturbed and control treatments, and a simultaneous increase of resistance and resilience in response to pulsed disturbances was hinted (Fig. 4; Table 1). In contrast, under limiting nutrient conditions (oDOM), only maximal growth rates but not RRN, %TF, or genome sizes increased significantly in response to disturbances (Fig. 5C; Table 1). Furthermore, no trade-offs between resistance and resilience were apparent in the presence of resource constraints (Fig. 5; Table 2). Taken together, our results indicate that generalist taxa with large genomes and elevated %TF only had competitive advantages under high resource availability in combination with pulse disturbances, suggesting the existence of metabolic costs related to these traits. This observation reflects results from the above-reported functional resistance measurements, where resource limitation constrained the resistance of BP (Fig. 4A).

Both resistant and resilient taxa should benefit from a temporally variable environment as the growth of resistant taxa is little impacted by environmental change and resilient taxa can recover fast. In line with this, the disturbance of communities with simultaneously highly resistant and resilient taxa has been predicted to lead to increased diversity because these communities can optimally exploit new niches that occur in temporally variable environments (16). In agreement with these theoretical predictions, we found that disturbances simultaneously increased the resistance and resilience of community members and resulted in significantly higher bacterial diversity compared to undisturbed controls when in the absence of resource limitation (Fig. 5J; Table 2). In contrast, disturbances did not select taxa with higher resistance, and species diversity did not differ from control treatments in communities grown under resource limitation (Fig. 5I; Table 2).

Shifts toward increased diversity and more resistant community members have both been debated as mechanisms that can increase community-level functional resistance to disturbances (10, 17). The molecular data presented previously provide evidence on the genomic traits and associated diversity structures that underlie the response of microbial communities to disturbances.

### Conclusions

In summary, the responses of resistance and resilience traits, diversity, bacterial production, and growth indicate a pivotal role of resource availability in determining the outcomes of disturbances. We suggest that in the presence of resources, modulations of growth can counteract the effects of disturbances, leading to apparent resistance and resilience despite large taxa turnover. On the other hand, when resources are limiting, minimum requirements for cellular respiration (reflected in BGE), putatively related to protein and RNA repair, seem to constrain the response space for bacterial communities facing disturbances. This work accordingly provides empirical evidence of the role of resource availability in determining the compositional and functional responses of bacterial communities to disturbances. Our results indicate community re-assembly toward resistant and resilient taxa with consequences for community diversity and functioning. However, this capacity to re-assemble was restricted by nutrient availability. While stochasticity was important during community re-assembly, we found consistent patterns of functional characteristics, genomic trait distribution and diversity patterns. Our findings suggest that selection occurred on traits rather than on individual populations, thereby leading to alternative compositional solutions with a similar functionality. This sheds new light on the notion of prevalent functional redundancy in microbial communities. A better understanding of the functional consequences and underlying mechanisms of microbial community resistance and resilience will be important considering the increasing frequencies, concomitance, and magnitude of multiple global change perturbations to ecosystems, including coastal marine areas. Future field studies should aim to validate the relevance of our findings within natural ecosystems (33).

## MATERIALS AND METHODS

### Starting communities and culture media

Microbial inocula for the continuous culture experiment were sampled from several sites in the south of the Gulf of Lion, in southern France, that feature contrasting environmental variability (Fig. 2; Table S1). Larger organisms, such as bacterivorous protists, were excluded by a pre-filtration step using a 0.8-µm pore size filter (47 mm mixed cellulose esters, Millipore, MA, USA). The pre-filtered communities from the sample sites were concentrated on a 0.2 µm pore size filter (47 mm cellulose, Millipore, MA, USA) and conserved using a protocol for cryopreservation of whole communities (34), and cryo-aliquots from all sites were pooled in culture media at the start of the experiment. The culture media were based on artificial seawater (ASW; salinity: 38 psu, pH 8) (35) and amended with DOM supplements as the sole carbon, nitrogen, and phosphorus source, with no vitamins added. Trace metals, Fe, and EDTA were added at a concentration 100-fold smaller than that originally published. Two complex DOM supplements that supported the growth of cell densities as typically found in oligotrophic conditions (oDOM) or meso- to eutrophic conditions (eDOM) were prepared from different aquatic environments and differed in dissolved organic carbon and nitrogen concentrations (Table S3), following instructions described elsewhere (34). eDOM media were additionally amended with the yeast extract (0.28 mg L^−1^; Sigma-Aldrich, St. Louis, MO, USA).

### Experimental design

Overall, the setup of the continuous culture system resembled that of an earlier continuous culture study (36). We assessed the effects of two disturbance regimes (undisturbed control and weekly salinity disturbance with +13 psu) under two different resource availability treatments (oDOM and eDOM), with each experimental treatment conducted in triplicate (totaling 12 continuous cultures, Fig. 1). We started the long-term experiment with a preculture that was set up in batch modes to allow cryopreserved bacteria to actively grow before turning to the continuous mode and using the inoculum detailed previously. The applied flow rate was set to approximate the generation time measured for prokaryote communities from the Mediterranean Sea (~2.75 days) (37). The incubations were kept at 18°C in the dark during the entire experiment. In total, six pulse disturbances were applied within 41 days of the continuous flow mode (Fig. 1; Table S4).

We regularly tested the fresh medium via flow cytometry for potential contamination, sampling it after it had passed the tubing system but just before the inlet into the continuous culture. While potential occasional contaminations were detected, these occurred mostly toward the experiment end, never exceeded 10^5^ cells per mL^−1^, and were minor compared to cell concentrations in the vessels (<6.6%, Table S5). Notably, in none of the eDOM treatments we could detect particle counts exceeding a threshold that we considered potential contamination (Fig. S5; Table S5). Sporadically occurring contaminations are therefore unlikely to explain the strikingly dissimilar communities among replicates from all treatments after the second experimental week (Fig. S2).

### Community assembly

Community aliquots for DNA extraction and downstream metabarcoding were sampled at least weekly from the continuous culture outflow. In total, we obtained DNA samples on 0.2-µm pore size filters (47 mm cellulose, Millipore, MA, USA) from 9 sample days (Fig. 1B; Table S4). Filters for DNA extractions were stored at −30°C until further processing.

DNA extractions were performed using a QIAamp DNA Mini Kit (QIAGEN, Hilden, Germany) and sent for 16 s rRNA gene amplicon sequencing using the primer pair 515yf–926r (38). Sequence processing was performed using the DADA2 pipeline for R (39). A total of 1,447 ASVs were identified and were taxonomically assigned using the Genome Taxonomy Database (40). The taxonomic and phylogenetic compositional structures of the communities were assessed using Bray–Curtis distances or the pairwise abundance weighted UniFrac metric (41), respectively. To evaluate the impact of nutrient and disturbance regimes as well as time on community assembly, we performed a PERMANOVA.

### Community functional measurements

Cell growth in the continuous culture was estimated by measuring cell densities via flow cytometry as detailed elsewhere (42).

Functional resistance of the continuous culture was estimated weekly through the measurement of the below-described bulk community metabolic rates before and 1 hour after each induced disturbance in the disturbance treatments, with simultaneous measurement of the controls (Table S4). BP was assessed via ^3^H-leucine incorporation (43). Bacterial respiration was quantified as the oxygen consumption in a 5-mL glass flask using a SensorDish reader (PreSens, Regensburg, Germany). BGE was estimated by dividing BP by the sum of BP and respiration.

We applied log-response ratios (lnR) (44) to quantify the relative change of the functional rate F before and after the pulse disturbance in the disturbed communities (lnR_D_) and in the corresponding controls where no disturbance was introduced (lnR_C_). We considered the difference between both ratios as the resistance index for function F (RI_F_), which is similar to the effect size measurement published previously by Osenberg and collaborators (45).


$${RI}_{F}=-\left|{lnR}_{C}-{lnR}_{D}\right|$$

The larger the deviation of RI_F_ from 0, the lower/higher the functional resistance/sensitivity. The absolute difference between lnR_D_ and lnR_C_ consequently results in small values for high resistance and large values for low resistance. The multiplication of this term by −1 allowed us to display the obtained RI values on a more intuitive scale where small values reflect low resistance and high values have high resistance.

To test the impact of culture conditions over time on bulk community functional resistances, we performed two-way repeated-measures analyses of variances (rmANOVAs) on the resistance indices (RI_F_) considering time and DOM level. We additionally fitted a linear mixed model (LMM) on the RI by DOM level to evaluate the direction of potentially detected trends over time for each of the DOM conditions separately.

### Genomic trait distributions and species diversity

Genomic traits related to resistance (genome size, %TF) and resilience (RRN, maximal growth rates delineated from codon usage biases) were predicted for each ASV, as suggested elsewhere (18, 22). This was done using the hidden state prediction option included in the PICRUSt2 software v2.4.2 (46) using either the PICRUSt2 default reference database (genome size, %TF, codon usage biases) or trait values from reference genomes stored in the rrnDB (RRN) (47). NSTI values given by the PICRUSt2 for each prediction (i.e., each ASV) indicate the phylogenetic distance of reference genomes that were used for prediction and are an indicator for the reliability of the predictions. Abundance-weighted NSTI values across our samples ranged from 0.011 to 0.128 (PICRUSt2 reference database) or from 0.025 to 0.179 (rrnDB) and indicate the presence of close relatives in the respective reference databases that were used for prediction. Furthermore, comparisons of the PICRUSt trait predictions against quantification of these trait values directly from shotgun metagenomes from 12 samples of our experiment indicated that the PICRUSt trait predictions were more precise than the corresponding metagenome obtained values (Fig. S6). Based on the reported NSTI values in combination with earlier detailed analyses on the depths of the phylogenetic signal of the inspected traits (18) and the performed comparisons with metagenome data (Fig. S6), we conclude that our trait predictions were robust.

CWMs of predicted genomic trait data for each sample were computed by multiplying the predicted trait values of each ASV by its relative abundance and adding up these weighted values. The Shannon diversity index was computed from the ASV compositional data to estimate species diversity. We performed two-way rmANOVAs to assess the effect of time and the disturbance regime on bacterial abundances, Shannon diversity, and predicted genomic traits in each of the DOM regimes. For predicted genomic traits, we also performed a one-tailed paired *t* test on the mean values of the genomic traits to test the specific *a priori* hypothesis that resistance and resilience values would both increase (and not decrease) in response to pulsed salinity disturbances.

## Data Availability

R-scripts and Unix shell scripts used for the data analyses have been published on GitHub (https://github.com/angelrainf/chemo.disturbances). Amplicon and metagenomic sequence data for this study have been deposited in the European Nucleotide Archive (ENA) at EMBL-EBI under accession numbers PRJEB55672 and PRJEB67923, respectively.

## SUPPLEMENTAL MATERIAL

Supplemental material msphere.00727-23-s0001.docx Supplemental methods and Fig. S1 to S6.

Supplemental tables msphere.00727-23-s0002.xlsx Tables S1 to S7.

ASM does not own the copyrights to Supplemental Material that may be linked to, or accessed through, an article. The authors have granted ASM a non-exclusive, world-wide license to publish the Supplemental Material files. Please contact the corresponding author directly for reuse.
