# Supplementary material for: The cost of adaptability: resource availability constrains functional stability under pulsed disturbances
Source: mSphere. 2024 Jan 11;9(2):e00727-23. doi: 10.1128/msphere.00727-23 (PMC10900906; doi:10.1128/msphere.00727-23)
Supplement: Supplemental material — Supplemental methods and Fig. S1 to S6. [file msphere.00727-23-s0001.docx]

**Supplementary Information**

The cost of adaptability: resource availability constrains functional stability under pulsed disturbances

Rain-Franco Angel^a,#,*^, Peter Hannes^b^, Pavan de Moraes Guilherme^a,c,d^ & Beier Sara^a,d^

^a^UMR 7621 Laboratoire d’Océanographie Microbienne, Observatoire Océanologique de Banyuls-sur-Mer, Sorbonne Université, Banyuls-sur-Mer, France

^b^River Ecosystems Laboratory, Ecole Polytechnique Federale de Lausanne, Lausanne, Switzerland

^c^Graduate Program in Ecology and Natural Resources (PPGERN), Laboratory of Phycology, Department of Botany, Universidade Federal de São Carlos, São Carlos, Brazil

^d^Department of Biological Oceanography, Leibniz Institute for Baltic Sea Research Warnemünde, Rostock, Germany

#Address correspondence to Angel Rain-Franco, angel.rain@limnol.uzh.ch

*Present address: Limnological Station, Department of Plant and Microbial Biology, University of Zurich, Kilchberg, Switzerland

**Supplementary Information includes:**

Supplementary methods

Figures S1 to S6

Table S1 to S7

References

**Supplementary methods**

**Starting communities**

Microbial inocula for the continuous culture experiment were obtained from cryopreserved microbial community aliquots that had been prepared as described previously [1]. In short, microbial communities were prefiltered using a 0.8 μm pore size membrane filter to exclude larger organisms, such as protists, and subsequently concentrated by filtering on a 0.2 μm pore size membrane filter. The filters with the concentrated cells were immersed in cold (4 °C) 1 ml culture medium containing 5% DMSO as cryo-protectant for 15 minutes, and then shock frozen in liquid nitrogen. These community aliquots were conserved at -80°C until the experiment start.

The aquatic environments from which the community aliquots had been sampled comprised several sites in the south of the Gulf of Lyon, South France. The Mediterranean field station SOLA and the coastal lagoons La Palme and Gruissan are characterized by contrasting environmental variability (Fig. 2; Table S1). Cryopreserved community aliquots from these sites were pooled to obtain a ‘super-diverse’ metacommunity, with the aim that selection mechanisms during downstream experimental treatments could act on a possibly large collection of species with complementary traits.

**Culturing media**

Culture media in this study were based on artificial seawater (ASW) [2]; adjusted to 38 g l^–1^ of salinity and pH 8. Trace metals, Fe, and EDTA were added 100-fold less concentrated than originally published. The ASW was amended with DOM supplements as the sole carbon, nitrogen, and phosphorus source, and no vitamins were added.

DOM supplements were prepared from particular material retained after GF/F filtration of water from seven different aquatic environments that differed in their trophic status as described elsewhere [1]. Based on several pre-tests, we combined these DOMs supplements into low- and high-nutrient DOM supplements (referred to as oDOM and eDOM, respectively, Table S3) that supported the growth of cell densities as typically found in oligo- to mesotrophic conditions (oDOM) or eutrophic conditions (eDOM). eDOM media were additionally amended with yeast extract (0.28 mg l^-1^; Sigma–Aldrich, St. Louis, MO, United States) to add a highly labile DOM compound. Complex and diverse DOM sources as the DOM supplements for oDOM and eDOM media have been discussed to support the assembly of diverse prokaryotic communities. The preparation of concentrated oDOM and eDOM supplements further allowed us to repeatedly and reproducibly prepare large volumes of media that were necessary for the long-term continuous culture experiment.

**Experimental design**

We started the long-term experiment with a preculture that was set up in batch modus. For this purpose, cryopreserved communities from SOLA, Gruissan, and La Palme were resuscitated in 6 l of ASW medium to which all seven DOM supplements were added, each of them in half concentration compared to oDOM and eDOM media used later. This preculture was incubated for three days at 18°C under agitation by a magnetic stirrer. Subsequently, this preculture was split into two equal volumes. We added another half portion of either the oDOM or the eDOM media supplements to each of the volumes, respectively, except for the yeast extract, and maintained these cultures for three more days in batch condition before distributing them into the continuous system. After these six days in batch mode, both precultures (oDOM and eDOM) were distributed into their respective set of 6 reactor vessels (chemostats), where each reactor vessel received 300 ml of the appropriate preculture (Fig. 1). The inflow of oDOM and eDOM media was activated to top up the vessels (total volume of 400 ml) and to start the continuous mode. From the six chemostats assigned to each DOM condition, three were used as control and three for the pulse disturbance treatment. The medium was pumped from 6 × 2 l bottles (three containing oDOM and three containing eDOM medium) into the culture vessels using a peristaltic pump (ISMATEC, Cole-Parmer GmbH, Wertheim, Germany), where each bottle fed one control and one disturbance chemostat, respectively. The applied flow rate of 70 μl min^-1^ was set to approximate generation time measured for prokaryote communities from the Mediterranean Sea (~2.75 day,[3]). The cultures were mixed constantly with magnetic stirrers and ventilated with venting filters (0.2 µm, 64 mm diameter, Midisart® 2000, Sartorius, Göttingen, Germany) attached to the lids. Syringes (50 ml) were connected to the culture vessels via stainless steel needles and were used either to add saturated salt solutions to the disturbance treatment chemostats or to sample small volumes of the medium for flow cytometry, salinity, and functional rate measurements. Medium volumes of approximately 200 mL were sampled from the continuous culture’s outflow from two consecutive days for DNA extractions. Overall, the setup of the continuous culture system resembled that used in an earlier continuous culture study [4]. We further added two polyethylene biochips (Mutag BioChip 25™, MUTAG, Chemnitz, Germany) into each vessel to provide a surface for biofilm formation. Cells attached to the biofilm that are not washed out can recolonize the medium and the biofilm on the Mutag BioChip 25™ may therefore serve as a spatial refuge. We decided to add these chips to lower the risk of a potential collapse of the continuous cultures during the long-term experiment.

After filling the reactor vessels, the cultures were stabilized in continuous mode for two days before the first pulse disturbance. Salt pulse disturbances were applied once per week to the disturbance treatments by adding ~18 ml of a saturated NaCl solution after the same volume was removed from the reaction vessels (+13 gl^-1^ NaCl). The exact volume to be added was calculated each time and for each disturbance treatment vessel from the salinity measured in the respective vessel just before the addition of the salinity pulse, to avoid a potential continuous raise of the salinity baseline and ensure that the final salinity after each peak did not exceed a salinity of ~54 gl^-1^ NaCl. Due to the continuous flow mode, the salt was diluted. After seven days, before the next pulse disturbance was introduced, the baseline salinity was approximately reached (Fig. 1B). In total, six pulse disturbances were applied within 41 days of continuous flow mode (Fig. 1B; Table S4).

The incubations were kept at 18°C in dark during the entire experiment. All components of the continuous culture system were autoclaved before the start of the experiment. We changed the medium and the tubing system every 3-4 days to avoid potential contamination. We further tested the medium regularly for potential contamination after it had passed the tubing system, just before the inlet into the continuous culture, via flow cytometry and after subtraction of particle counts in freshly prepared sterile medium. The particle count data were distributed approximately symmetrically around 0 and Kolmogorov-Smirnov tests revealed no deviation of the particle count data in the tested media from a normal distribution with a mean of 0 (Fig. S5). The preparation of DOM supplements from GF/F filtered cell debris as detailed above causes the presence of small organic particles in the media and particle count data >0 may accordingly be due to noise rather than due to living contaminants (Fig. S5). We though cannot exclude that occasionally also true contaminations occurred. During our experiment, we considered more than 250 particle counts per second in the flow cytometer as a possible contamination, whereupon the affected tubing system and medium were replaced. This value corresponds depending on the daily measured flowrate of the flow cytometer to approximately 55500 particle counts per mL. Particle counts that surpassed this threshold and that were considered as potential contaminations were detected mostly towards the experiment end and never exceeded 10^5^ particles per mL (Table S5).

**Community assembly**

The samples taken for DNA extraction and downstream metabarcoding from the continuous culture outflow were filtered onto 0.22 μm filters (cellulose filters, Millipore, MA, United States) (Table S4). To obtain a sufficiently large water volume, a sterile water bottle was attached to the outflow approximately 48 h before sampling. Sampling was performed at least once per week. In week 3, an additional set of samples was obtained as the continuous flow had accidentally stopped for some hours in two vessels (vessels 2 and 5; Fig. 1B). Also, in week 6 two additional sets of samples for DNA were obtained (Table S4). In total, we acquired DNA samples from nine sample days (Fig. 1B). Filters for DNA extractions were stored at -30°C until further processing.

DNA extractions were performed using a QIAmp DNA Mini Kit (QIAGEN, Hilden, Germany) with an initial bead-beating step in ATL buffer using a FastPrep-24™ 5G (MP Biomedical, California, USA). The concentration and quality of the eluted DNA were tested using a DS11-FX+ microvolume spectrophotometer (DeNovix, Delaware, USA).

DNA samples from the continuous culture were sent for 16S rRNA gene amplicon sequencing (300 base pairs paired-end read, Illumina Miseq V3). The PCR amplification was performed by the sequencing company (LGC Genomics GmbH) using the primers pair 515yf-926r [5]. Libraries were demultiplexed by the sequencing company using the BCL2FASTQ software v2.17.1.14, which removed reads with length <100 bases and performed primer clipping (up to three mismatches per primer). Sequences were further processed using the DADA2 package [6] in R by slightly modifying the standard pipeline (truncLen=c(250,180)) [1]. A total of 1447 amplicon sequence variants (ASVs) were identified across samples. Retrieved ASVs were taxonomically assigned using the Genome Taxonomy Database (GTDB) [7]. Read count data were rarefied to the minimum number of reads obtained for a sample across all samples (9,412 reads). See Table S6 for all downstream data on community compositional data (beta diversity and alpha diversity dependent analyses, genomic trait distributions).

**Beta nearest taxon index (****βNTI)**

We computed the βNTIs to evaluate assembly mechanisms [8, 9] in response to the disturbance regimes under the different DOM levels. The βNTI assesses the governing assembly mechanism which will drive the phylogenetic similarity (or dissimilarity) between a pair of samples, relative to the expectation from a null model derived from a reference species pool. A phylogenetic similarity surpassing the theoretical expectation βNTI >2 indicates the prevalence of variable deterministic assembly processes during community succession. A phylogenetic similarity below the theoretical expectation βNTI< -2 indicates the prevalence of homogeneous deterministic assembly processes during community succession. Finally, βNTIs between -2 and 2 indicate that community assembly is driven by stochastic processes rather than deterministic processes. βNTIs were calculated via the iCAMP R-package (v1.3.4) [10]. βNTIs during community succession were estimated by comparing communities from each chemostat vessel to the community of same vessel from the respectively following sampling time. The null models to obtain βNTIs were estimated relative to the total species pool from all incubations under the same DOM level and same disturbance regime over the incubation (=27 samples). This reference species pool was chosen because we aimed to focus on assembly mechanisms under the respectively relevant disturbance and DOM regime over time.

**Community functional measurements**

Cell growth in the continuous culture was estimated by measuring cell densities via flow cytometry as detailed elsewhere [11]. Briefly, aliquots (1350 µl) were sampled using sterile syringes, fixed with glutaraldehyde (0.1% final concentration), and stored at -80°C. Samples were analyzed in the Cytoflex Flow Cytometer (Beckman Coulter, California, USA). Cell counts per mL medium were computed after substracting particle count values from freshly prepared sterile medium as blank value.

Functional resistance of the continuous culture was estimated weekly by measuring the below-described bulk community functional rates before and 1 hour after each induced disturbance in the disturbance treatments and simultaneously also in the controls (Table S4). Heterotroph bacterial production (BP) was assessed via ^3^H-leucine incorporation: 100 μl of a working solution containing 1 part ^3^H-leucine (125.6 Ci mmol^–1^, Perkin Elmer ^TM^, Massachusetts, USA) and 4 parts cold leucine were added to 1.5 ml sample water (final leucine concentration: 40 nM) and incubated in dark at 18°C. The incubations were stopped after 1.5 hours by adding 150 μl of 50% trichloroacetic acid. For each reactor vessel and measure point, two technical replicates and one blank control that was stopped by adding 50% trichloroacetic acid before the incubation started were performed. The incubations were then processed using the microcentrifuge method as published elsewhere [12], and quantified using a liquid scintillation counter (Hidex 300SL HIDEX, Turku, Finland). The theoretical conversion factor of 1.55 kg C mol^−1^ was used to convert leucine incorporation rates to carbon production [13].

Respiration was quantified as the oxygen consumption estimated in 5 ml glass vials equipped with an OxoDish using a SensorDish reader (PreSens, Regensburg, Germany). The tubes were filled with sample water and closed with an air-tight lid while avoiding the enclosure of air bubbles. The filled vials were subsequently placed in an incubator at 18°C in dark. Respiration was estimated as the rate of oxygen decrease from a linear fitting from oxygen measurements taken every 3 min during 14 h. Bacterial growth efficiency (BGE in %) was estimated by dividing BP by the sum of BP and respiration, while the respiration rates were transformed to carbon consumption considering a respiratory quotient of 0.89 [14].

**Resistance index**

Log-response ratios (lnR) are widely used metrics to measure the relative impact of a treatment on a given variable, independently of its metric units [15]. In this study, we applied the lnR [16] to quantify the relative change of the functional rate F before and after the pulse disturbance in the disturbed communities (lnR_D_) and in the corresponding controls where no disturbance was introduced (lnR_C_) at the same time intervals. For the lnR_C_, the average of the triplicates was calculated to cover the total range of variability of the response variable.

Values of the lnR close to 0 represent a low variability of the measured functional rate, while deviations from 0 indicate an increase (decrease) of functional rates reflected in positive (negative) lnR during the interval of consideration. We considered the difference between both ratios as the resistance index for F (RI_F_) which is similar to the effect size measurement published previously by Osenberg and collaborators [17]. Differently from the originally published metric, we used absolute difference since we were interested in a resistance metric that was independent of the direction of change. We also did not normalize by time, as the considered time interval was equal for lnR_D_ and lnR_C_.

$${RI}_{F}=-\left| {lnR}_{C}-{lnR}_{D} \right|$$

If RI_F_ approximates zero, the temporal change of the functional response F was similar in magnitude and direction in both the disturbed and control communities. In this case, F was only marginally impacted by the induced salinity pulse pointing to a high resistance level. The larger the deviation of RI_F_ from zero, the lower/higher the functional resistance/sensitivity. The absolute difference between lnR_D_ and lnR_C_ consequently results in small values for high resistance and large values for low resistance. The multiplication of this term by -1 allowed us to display the obtained RI values on a more intuitive scale where small values reflect low resistance and high values have high resistance.

**Genomic trait distributions from PICRUSt preditions and species diversity**

Genomic traits that are indicative of the life history of prokaryotes feature significant phylogenetic signals and can be extrapolated from taxonomic marker genes of fully sequenced closely related relatives for the uncultured species in a community [18]. Genomic traits were predicted for each ASV using the hidden state prediction option included in the PICRUSt2 v.2.4.2, [19]. This was done based on recently published trait values of strains included in the default PICRUSt2 reference tree for minimal generation time, percent transcription factors (%TF), and genome size [18]. In order to obtain values for ASV specific maximal growth rate predictions, we used the reciprocal minimal predicted generation times.

Due to possibly problematic values for the number of 16s rRNA gene copies (RRN) of the internal PICRUSt2 RRN reference database [18] we instead predicted RRN using trait values available via the Ribosomal RNA Operon Copy Number Database (rrnDB) [20]. For this purpose, rrnDB 16s rRNA gene sequences (rrnDB v5.7) were aligned with the MUSCLE software [21] and used to construct a phylogenetic tree via the FastTree software (vs 2.1.10, GTR substitution model, gamma distribution) [22]. The output phylogenetic tree was integrated as backbone phylogeny into PICRUSt2 to predict RRNs for all ASVs in our sample based on curated RRN values from rrnDB as the reference database. The average phylogenetic distances to the closest relatives in the PICRUSt2 default reference database ranged from 0.011-0.128 for the internal reference database integrated into the PICRUSt2 software and from 0.025-0.179 for the rrnDB (abundance weighted NSTI, Table S7).

Community weighted means (CWMs) of genomic trait data in each sample were computed by multiplying the predicted trait values for each ASV with its relative abundance and adding up these weighted values in each sample.

The predictions for minimal generation time and accordingly also maximal growth rates were based on the codon usage bias as detailed elsewhere [23]. Weissmann et al. suggested that only generation times up to 5 h can be reliably predicted from the codon usage bias, while the prediction of larger generation times becomes increasingly inaccurate. CWMs of generation times in our samples were in most cases ≤ 5h and reached maximally 6.2 h. We, therefore, decided that generation time estimations were sufficiently accurate.

In agreement with earlier considerations, we classified RRN and generation time as resilience-related traits and %TF and genome size as resistance-related traits [24]. The Shannon diversity index was computed from the ASV compositional data to estimate species diversity.

**Genomic trait distributions from shotgun metagenome samples**

The extracted DNA from day 41 at the experiment end was sent for DNA shotgun metagenome sequencing (DNA shotgun library, insert size: 300 bp, 2 × 150 bp reads, Illumina NextSeq 500 V2) in order to compare amplicon enabled PICRUSt trait predictions to metagenome enabled estimations of trait CWM values. Metagenome paired-end reads were quality trimmed using the sickle software v1.33 (q= 20, minimum length=75). The quality trimmed reads were assembled using the metaSpades assembler v3.13.0 (settings: k-mers from 21 to 99, with 4 nucleotide steps). Genes prediction was performed via the prodigal software v2.6.3. Protein-coding reads were mapped on the contigs with predicted open reading frames from the assembly using the bowtie2 v2.3.4.3 software (settings: very-sensitive-local) and summarized with the featurecounts software v1.4.6-p2 . Functional annotation of the predicted genes was done via diamondblast v0.8.22 using the KEGG database (downloaded May 2016) with e-value cutoff of 1e^-5^. Predicted genes were furthermore functionally annotaded against the PFAM database with the interproscan software (v5.62-94.0) .

We used the microbecensus software v1.1.1 to predict the average genome size of all sequenced genomes in each community from the quality trimmed reads, where the resulting average genome size corresponds to the CWM. The CWM for RRN was estimated after determining the number of quality trimmed reads encoding the 16s rRNA gene with the SortMeRNA software (v4.3.6; settings: fastx=true, other=false, no-best=false) against the silva-arc-16s-id95.fasta and the silva-bac-16s-id90.fasta databases. The number of reads encoding the 16s rRNA genes (after normalisation by average reads lengths and the lengths of the *E. coli* 16s rRNA gene) per genome equivalent was used as value for the CWM of RRN. The number of sequenced genome equivalents is an output from the microbecensus software and indicates the number of genomes covered by the specific sequence depths of a sample.

We applied the gRodon R package to predict the average maximal growth rate of each genome in the community. For this purpose we used predicted genes encoding ribosomal genes (KEGG pathway 03010) as highly expressed genes. The contig abundances were used as argument for the coverage option of the predictGrowth function (gRodon software) and were estimated analogously to the transcript per million metric as gene copies per million as detailed elsewhere [25]. The mode option of the predictGrowth function was set to “metagenome_v2”. The predictGrowth function output includes an estimation of the average (i.e. CWM) minimal generation time and we used the reciprocal of this value as average maximal growth rate.

PFAM annotations were used to identify a shortlist of potential transcription factors. A value for the CWM of %TF was obtained from the sum of relative gene abundances of each predicted transcription factor based on the gene copy per million metric that was divided by 10000 to obtain a percent value (instead of per million).

The robustness of PICRUSt trait predictions was evaluated by plotting PICRUSt predicted trait values against those obtained from shotgun metagenome sequencing as detailed above (Fig. S6).

**Statistical analysis**

Statistical analyses were performed in R [26]. At the end of the long-term experiment (day 41), each replicate of the disturbance treatment under eDOM and oDOM was affected by a sudden community collapse (Fig. 4D). We have therefore considered data obtained from flow cytometry for statistical analyses only until day 40. Data obtained from the final DNA sampling event on day 41 were included in the downstream statistical analyses as it was sampled early during day 41 and most of the water volume was derived from day 39 or 40.

Compositional changes of the communities were evaluated using the Bray-Curtis distance and the phylogenetic compositional structure of the communities was assessed using a pairwise abundance weighted UniFrac metric [27]. To test the effect of time, disturbance treatment, and DOM regime on the (phylogenetic) community composition, we performed permutational multivariate analyses of variance (PERMANOVA) using the function adonis2 available in the “vegan” R-package (permutations=1000)[28].

To assess the hypothesis of the gained functional resistance under high resource conditions we performed two-way repeated-measurements analyses of variances (rmANOVAs) on the resistance indices (RI_F_) considering time and DOM level. We additionally fitted mixed linear models to evaluate the direction of potentially detected trends over time, using the “nlme” R-package [29]. For this model, we considered time as a fixed factor and the replicates as random factors.

We furthermore performed two-way rmANOVAs to assess the effect of time and the disturbance regime on bacterial abundances, Shannon diversity, and genomic traits in each of the DOM regimes, separately. In our analysis, we aimed to test the one-tailed hypotheses predicting a simultaneous increase of resistance and resilience levels and the associated genomic trait of aquatic prokaryote communities in response to disturbances [24]. More precisely, we expected higher RRN, higher %TF, higher maximal growth rates, and higher RRN in disturbed compared to undisturbed treatments. As a consequence of formulating one-tailed hypotheses, we performed one-tail paired t-tests on the mean values of the genomic traits in addition to the rmANOVAs to test if the a priori hypothesized direction of the response values was observed. Normality and homogeneity of variance of the data were tested by the Kolmogorov-Smirnov and the Levene test, respectively. Overall, no violations of the ANOVA assumptions concerning normality and homogeneity of variance were detected, except for bacterial abundance and community respiration. In these cases, a square root transformation was applied to the data to fulfill these assumptions.

**Fig. S1. Salinity variations at the sites sampled for source communities.** A) Time series salinity dynamics at the three sites La Palme and Gruissan Lagoons and SOLA stations fitted via loess smoothing. B) Plot displaying measured salinity differences during all pairwise sampling intervals (days). Time series data for La Palme (23/09/1989-11/09/2020, n = 206) and Gruissan (23/09/1989-08/08/2019, n = 128) were retrieved from https://wwz.ifremer.fr/surval. Environmental data for the SOLA station was provided by the SOMLIT program (https://www.somlit.fr/; 04/01/2005-24/11/2020, n = 1443).

**Figure S2. Community dynamics.** Relative abundance of the 10 most abundant orders in the continuous culture communities at **A)** oDOM and **B)** eDOM levels at each sampling day. The numbers above the graphs refer to the replicates of each treatment.

**Figure S3 Influence of deterministic versus stochastic processes on microbial community dynamics.** The influence of deterministic versus stochastic processes on microbial community dynamics was quantified during the course of the continuous culture experiment via null model analyses. For this purpose, we applied the βNTIs [8, 9] separately for disturbance and DOM regimes and over time applying a sliding window setup in the continuous cultures and using the iCAMP R-package (v1.3.4) [10].

The βNTI evaluates whether the phylogenetic similarity between a pair of samples is significantly lower or higher than expected by chance relative to a reference species pool. Phylogenetic similarity surpassing the theoretical expectation (βNTI >2) indicates the prevalence of variable deterministic assembly processes during community succession. Phylogenetic similarity below the theoretical expectation (βNTI< -2), indicates the prevalence of homogeneous deterministic assembly processes during community succession. βNTIs between -2 and 2 indicate that community assembly is driven by stochastic rather than a deterministic processes.

For our analyses βNTIs during community succession were estimated in a way that communities from each chemostat vessel were compared to the community of same vessel from the respectively following sampling time. The null models to obtain βNTIs were estimated relative to the total species pool from all incubations under the same DOM level and same disturbance regime over the incubation (=27 samples). This reference species pool was chosen because we aimed to focus on assembly mechanisms under the respectively relevant disturbance and DOM regime over time.

**Figure S4.** **Community structures.** A) Overview PCoA biplot including all data points (weighted Unifrac distances). B) PcoAs (weighted Unifrac distances) for individual sampling days; axes of the individual plots are differently scaled as indicated by the grid lines.

**Figure S5. Distribution of the potential contaminations in the feeding media.**

The boxplots represent data points from flow cytometry measurements of the medium in the tubing outlet that was tested regularly for potential contaminations (Table S5). Detected particle numbers corresponded to values obtained after subtraction of the blank control (sterile medium). Kolmogorov-Smirnov (KS) tests did not indicate a deviation of the data points from a normal distribution of mean=0 and standard deviation =1( P-values>0.05). The resulting approximate symmetrical distribution of data points around 0 suggests that data points >0 may not be true contaminations, but rather noise. This is particularly the case because the preparation of DOM supplements from GF/F filtered cell debris causes the presence of small organic particles in the media. During our experiment, we considered more than 250 particle counts per second in the flow cytometer as a possible contamination, whereupon the affected tubing system and medium were replaced. These value corresponds depending on the daily measured flowrate of the flow cytometer to approximately 55500 particle counts per mL and the respective values are highlighted by filled circles.

**Figure S6. PICRUSt versus metagenome trait predictions**

To test the robustness of PICRUSt trait predictions we plotted PICRUSt predicted trait values against those obtained from shotgun metagenome sequencing that were available for the communities of all 12 chemostat vessels at day 41: A) Genome size, B) Fraction of transcription factors (%TF), C) Maximal growth rate (µmax) and D) number of 16s rRNA gene copies (RRN).

The two pink data points indicate communities from oDOM disturbed, 2^nd^ replicate and eDOM control, 3^rd^ replicate, which both were dominated by the ASV SV_10 affiliating with the Sphingomonadales family (Fig. S2). Pink and black values indicate the output from Pearson correlations with all data points (pink) or after removing the pink data points (black).

Different than genome size and RRN, it is not possible to quantify %TF and µmax directly from metagenome reads, while, only about 80% of all metagenome reads could be mapped back onto the assembly. However, rare species are typically overrepresented among reads that cannot be assembled and simultaneously, life history strategies may not be equally distributed among the rare and the abundant biosphere. For this reason, the metagenome based estimates of %TF and µmax might be biased. In the case of µmax additional biases can be assumed, because µmax is delineated from codon usage biases. This is a measure of within-genome differences in codon utilization, while the affiliation of contigs to certain genomes is lost in metagenomes and not considered in the metagenome_v2 mode of the gRodon package. The metagenome_v2 mode is therefore less accurate than predictions for individual genomes.

The resulting correlations between metagenome-based estimates and PICRUSt predictions were for none of the genomic traits significant. However, after removing two outlier samples (highlighted in pink), we found pronoucedly significant correlations for genome size and RRN (r=0.86, p=0.0001 and r=0.69, p=0.0028, respectively), while %TF and RRN remained with poor correlations. After in-depth inspections of metagenome and PICRUSt-based estimates, we concluded that the two outlier samples were most likely induced by biased metagenome predictions while we consider the PICRUSt predictions to be of superior robustness. Please see our argumentation as detailed below:

1. The two outlier samples were dominated by one ASV (SV_10, ~50% abundance in both outlier samples) affiliating with the Sphingomonadales family that in the remaining 10 samples occurred only at low abundance (Fig. S2). We therefore assumed that mismatches of the two outlier samples were caused by the species being represented by this ASV. Mismatches of metagenome versus PICRUSt genomic trait predictions for this ASV could be due to (i) relatives in the PICRUSt reference database are not close enough for robust predictions; (ii) erroneous genomic trait values of reference species that were used for the PICRUSt prediction and (iii) erroneous microbecensus genome size prediction in metagenomes in chemostat samples.
2. In a recent publication [24] (See figure S5C in there) genome size trait values from representatives of the PICRUSt reference database obtained from IMG estimates (https://img.jgi.doe.gov/, assembly based) were compared against values obtained from individual reads of these genomes using the microbecensus software. While there was a close match between genome sizes from IMG and those determined by microbe census for most genomes, in about 1% of the inspected genomes a larger deviation between microbecensus and IMG estimates was observed. For this analysis only genomes with >2,000,000 sequenced reads and >50 sequenced genome equivalents were considered. Biased IMG or microbecensus genome size estimates due to low sequence depths can therefore be excluded as reason for mismatches between the two genome size estimates. However, microbecensus estimates genome sizes based on the fraction of total reads mapping to obligatory single copy genes. If such single copy genes are exceptionally missing or are duplicated in a genome the microbecensus genome size output value will over- or underestimate the true genome size.
3. In case of (i) outliers should occur in the correlations for genome size, %TF and µmax as in these cases the same reference species from the default PICRUSt reference database was used for prediction. RRN was predicted from the rrnDB with other reference species, and outliers samples may not necessarily occur as outliers in the RRN regression (Fig. S6 D). However, we observed the occurrence of outlier samples for genome size and RRN, while these samples did not appear as outliers for %TF and µmax.
4. In case of (ii) outliers that occurred for the genome size correlations should also occur in %TF correlations. This is because %TF for species in the reference database was calculated by dividing the number of genes encoding transcription factors by the assembly-based IMG genome size. Accordingly, if there was a bias in the genome size of the reference species used to predict the Sphingomonas ASV SV_10, this should have also affected the %TF estimate. The outlier samples should instead not occur in the µmax or RRN correlations.
5. In case (iii) outliers should occur in correlations for genome size and RRN, but not in the µmax or %TF correlations. This is because the metagenome-based genome size and RRN prediction both depend on the microbecensus output, while metagenome-based %TF and µmax predictions are independent of the microbecensus output. This is the scenario that we observed. The specific occurrence pattern of outliers therefore indicates that the microbecensus software produced a biased value for average genome size (and accordingly also RRN) in the two outlier samples, possibly because of exceptionally missing or duplicated ‘obligatory single copy genes’ in the genome representing SV_10. All remaining samples provided a high correlation for both genome size and RRN. Low correlation for %TF and µmax independent from the removal of outliers were expected as detailed above.
6. We further inspected the genome sizes of 14 genomes affiliating according to the GTDB phylogeny (https://gtdb.ecogenomic.org/ ) with the same species as SV_10 (Alteriqipengyuania bathyomarina). The PICRUSt predicted genome size of SV_10 was 3.15 Mbp, while the genome sizes of GTDB genomes affiliating with the same species was 3.13±0.29 Mbp (mean±stdv). The deviation of the predicted SV_10 genome size from the mean of the 14 GTDB genomes was accordingly 0.76% and the standard deviation of within-species genome sizes represented <10% of the mean value. This indicates a reasonable and robust genome size prediction for SV_10 via the PICRUSt software.

**References**

1. Rain-Franco A, de Moraes GP, Beier S. Cryopreservation and Resuscitation of Natural Aquatic Prokaryotic Communities. *Frontiers in Microbiology* 2021; **11**: 3633.

2. Eguchi M, Nishikawa T, Macdonald K, Cavicchioli R, Gottschal JC, Kjelleberg S. Responses to Stress and Nutrient Availability by the Marine Ultramicrobacterium Sphingomonas sp. Strain RB2256. *Appl Environ Microbiol* 1996; **62**: 1287.

3. Landa M, Cottrell MT, Kirchman DL, Blain S, Obernosterer I. Changes in bacterial diversity in response to dissolved organic matter supply in a continuous culture experiment. *Aquat Microb Ecol* 2013; **69**: 157–168.

4. Baho DL, Peter H, Tranvik LJ. Resistance and resilience of microbial communities - temporal and spatial insurance against perturbations. *Environ Microbiol* 2012; **14**: 2283–2292.

5. Parada AE, Needham DM, Fuhrman JA. Every base matters : assessing small subunit rRNA primers for marine microbiomes with mock communities , time series and global field samples. 2016; **18**: 1403–1414.

6. Callahan BJ, Mcmurdie PJ, Rosen MJ, Han AW, Johnson AJA, Holmes SP. DADA2 : High-resolution sample inference from Illumina amplicon data. 2016; **13**.

7. Parks DH, Chuvochina M, Waite DW, Rinke C, Skarshewski A, Chaumeil P-A, et al. A standardized bacterial taxonomy based on genome phylogeny substantially revises the tree of life. *Nature Biotechnology* 2018; **36**: 996–1004.

8. Webb CO. Exploring the Phylogenetic Structure of Ecological Communities: An Example for Rain Forest Trees. *The American Naturalist* 2000; **156**: 145–155.

9. Stegen JC, Lin X, Konopka AE, Fredrickson JK. Stochastic and deterministic assembly processes in subsurface microbial communities. *The ISME Journal* 2012; **6**: 1653–1664.

10. Ning D, Yuan M, Wu L, Zhang Y, Guo X, Zhou X, et al. A quantitative framework reveals ecological drivers of grassland microbial community assembly in response to warming. *Nature Communications* 2020; **11**: 4717.

11. Marie D, Simon N, Guillou L, Partensky F, Vaulot D. Flow Cytometry Analysis of Marine Picoplankton. In: Diamond RA, Demaggio S (eds). *In Living Color: Protocols in Flow Cytometry and Cell Sorting*. 2000. Springer, Berlin, Heidelberg, pp 421–454.

12. Smith D, Azam F. A simple economical method for measuring bacterial protein synthesis rates in seawater using 3H-Leucine. *Marine Microbial Food Webs* 1992; **6**: 107–114.

13. Kirchman D. Handbook of Methods in Aquatic Microbial Ecology. 1993.

14. Williams PJ le B, Giorgio PA del. Respiration in aquatic ecosystems: history and background. *Respiration in Aquatic Ecosystems* . 2005. Oxford University Press.

15. Hedges LV, Gurevitch J, Curtis PS. The Meta-Analysis of Response Ratios in Experimental Ecology. *Ecology* 1999; **80**: 1150–1156.

16. Hillebrand H, Gurevitch J. Meta-Analysis and Systematic Reviews in Ecology. In: John Wiley & Sons Ltd (ed). *eLS*. 2016. John Wiley & Sons, Ltd, Chichester, UK, pp 1–11.

17. Osenberg CW, Sarnelle O, Cooper SD. Effect Size in Ecological Experiments: The Application of Biological Models in Meta‐Analysis. *The American Naturalist* 1997; **150**: 798–812.

18. Beier S, Werner J, Bouvier T, Mouquet N, Violle C. Trait-trait relationships and functional tradeoffs vary with genome size in prokaryotes. *bioRxiv* 2021; 2021.07.23.453341.

19. Douglas GM, Maffei VJ, Zaneveld JR, Yurgel SN, Brown JR, Taylor CM, et al. PICRUSt2 for prediction of metagenome functions. *Nature Biotechnology* 2020; **38**: 685–688.

20. Stoddard SF, Smith BJ, Hein R, Roller BRK, Schmidt TM. rrnDB: improved tools for interpreting rRNA gene abundance in bacteria and archaea and a new foundation for future development. *Nucleic Acids Res* 2015; **43**: D593–D598.

21. Edgar RC. MUSCLE: a multiple sequence alignment method with reduced time and space complexity. *BMC Bioinformatics* 2004; **5**: 1–19.

22. Price MN, Dehal PS, Arkin AP. FastTree 2-Approximately Maximum-Likelihood Trees for Large Alignments. *PLoS One* 2010; **5**.

23. Weissman JL, Hou S, Fuhrman JA. Estimating maximal microbial growth rates from cultures, metagenomes, and single cells via codon usage patterns. *PNAS* 2021; **118**.

24. Beier S, Werner J, Bouvier T, Mouquet N, Violle C. Trait-trait relationships and tradeoffs vary with genome size in prokaryotes. *Frontiers in Microbiology* 2022; **13**.

25. Wagner GP, Kin K, Lynch VJ. Measurement of mRNA abundance using RNA-seq data: RPKM measure is inconsistent among samples. *Theory Biosci* 2012; **131**: 281–285.

26. R Core Team. R: A Language and Environment for Statistical Computing. 2018. R Foundation for Statistical Computing, Vienna, Austria.

27. Lozupone CA, Knight R. Species divergence and the measurement of microbial diversity. *Fems Microbiology Reviews* 2008; **32**: 557–578.

28. Oksanen J, Blanchet FG, Friendly M, Kindt R, Legendre P, McGlinn D, et al. vegan: Community Ecology Package, R package version 2.5-6. 2019.

29. Pinheiro J, Bates D, DebRoy S, Sarkar D, R Core Team. {nlme}: Linear and Nonlinear Mixed Effects Models. 2020.
